# Prevalence, Awareness, Treatment, and Control and Related Factors of Hypertension in Multiethnic Agriculture, Stock-Raising, and Urban Xinjiang, Northwest China: A Cross-Sectional Screening for 47000 Adults

**DOI:** 10.1155/2019/3576853

**Published:** 2019-11-03

**Authors:** Lin Wang, Nanfang Li, Mulalibieke Heizhati, Xiaoguang Yao, Gulinuer Duiyimuhan, Keming Zhou, Mei Cao, Menghui Wang, Junli Hu, Delian Zhang

**Affiliations:** Hypertension Center of People's Hospital of Xinjiang Uygur Autonomous Region, Hypertension Institute of Xinjiang, Urumqi 830001, Xinjiang, China

## Abstract

**Background:**

Hypertension is the leading cause of cardiovascular disease. Distribution of hypertension and related factors among multiethnic population in Northwest China remains scarce. The aim was to determine prevalence, awareness, treatment, control, and risk factors associated with hypertension among multiethnic population in Northwest China.

**Methods:**

We conducted a blood pressure (BP) screening project covering a third of adults in Emin Xinjiang, Northwest China, during 2014–2016. Hypertension was defined as systolic BP ≥ 140 mmHg, diastolic BP ≥ 90 mmHg, and/or taking antihypertension drugs. We compared prevalence, awareness, treatment, and control of hypertension and related factors by different regions (agriculture, stock-raising, or urban) and by ethnic groups.

**Results:**

Totally 47,040 adults were screened with 48.5% women. Overall prevalence, awareness, treatment, and control of hypertension were 26.5%, 64.6%, 44.5%, and 15.3%, respectively. Age-gender-adjusted hypertension prevalence was higher in urban (28.2%) than in other regions and in Kazakh (30.3%) than in others. The lowest awareness and treatment rates were observed in the agricultural region and in Kazakh subjects, while the lowest control was in the stock-raising region (13.8%) and in Kazakh subjects (12.6%). After adjusting for age, gender, ethnicity, and regions, compared to normal weight, nonsmokers, and nondrinkers, obesity, smoking, and alcohol intake were significantly related to increased prevalence of hypertension by 94%, 1.5, and 3.9 folds, respectively.

**Conclusions:**

Disparities in hypertension control among regions and ethnic groups suggested inadequate screening and treatment, especially in stock-raising regions and Kazakh populations. Control of alcohol intake, smoking, and obesity should be at high priority of health promotion.

## 1. Introduction

Globally, cardiovascular disease (CVD) including stroke leads to 17 million deaths each year, of which 80% occurs in low- or middle-income countries such as China [1, 2]. Based on nationwide data in 2013-2014, the age-standardized prevalence, incidence, and mortality rates for stroke were 1114.8, 246.8, and 114.8 per 100,000 population in China [3]. Furthermore, in some regions such as Xinjiang, these rates are even higher (1176.4, 316.2, and 138.5 per 100,000 population) [3]. Over 70% of the stroke burden can be attributed to hypertension in China [2], and over 80% of stroke survivors in northwest regions encompassing Xinjiang are reported to have hypertension [4]. As the leading risk factor of CVD, hypertension is highly prevalent in Xinjiang, affecting 35.01% of adults aged ≥35 years and 40.7% of those ≥45 years [5, 6].

Xinjiang, a relatively undeveloped province with a multiethnic and multicultural population, is one of the four stock-raising areas (accounting for 34.68% of the total land) in China and is bordered with Central Asian countries. One-fifth of local residents are leading nomadic or seminomadic lives, making the access of fresh vegetables and fruits difficult, the penetration rate of medical resources low, and poor health awareness poor [7]. All these factors may lead to a higher prevalence of hypertension and its poor management especially among residents in the stock-raising region. Nonetheless, information about the prevalence, awareness, treatment, and control rates and the contributing factors of hypertension in the stock-raising region is limited. It is well known that hypertension is a complex interaction of multiple genetic, environmental, and behavioral factors [8, 9]. The timely exploration of the magnitude and management of hypertension is fundamental.

Therefore, this study aimed to explore the regional and ethnic disparities in hypertension status and to in-depth analyze potential variations in prevalence, awareness, treatment, and control of hypertension and associated factors. Thereby, the results may provide basis for the design and implementation of appropriate interventions for hypertension in the Xinjiang area extending to the Central Asian countries with the same ethnicity and approximate conditions.

## 2. Materials and Methods

### 2.1. Study Population

All the participants were selected from the hypertension screening project which included health archives, health check-up records, and disease registrations with a solid information security system from 1 Jan 2014 to 31 Dec 2016 in Emin, Xinjiang. Emin is one of the counties with a larger population base in Xinjiang, including 4 towns, 7 townships, and 6 stock-raising regions. There are 25 ethnic groups with four main groups such as Han, Kazakh, Uygur, and Mongolian, and with a total population of over 160,000. Of them, population aged ≥15 accounts for 83.36%. In addition, Han and Uygur residents mainly live in the urban and agricultural setting, while Kazakh and Mongolian residents in the stock-raising setting.

### 2.2. Data Collection

A community-based door-to-door screening for the health behavior questionnaire and anthropometric examination were conducted. Eligible population included residents aged ≥15 years and living ≥6 months. A unified questionnaire was taken including questions on demographics, educational status, cigarette consumption, alcohol intake, and hypertension-related information (Whether it was previously diagnosed by a doctor? Whether it has been being treated? Whether he/she is taking antihypertensive drugs within the previous two weeks?). The Hypertension Center of the People's Hospital of Xinjiang Uygur Autonomous Region designed the survey, and it was approved by the Ethics Committee of the abovementioned hospital. Informed consent was obtained from all participants. Participant's anonymity was accomplished by using a nontraceable ID-number. Anthropometric variables were measured using standard equipment and procedures.

The blood pressure (BP) values of the individuals were obtained with three measurements taken after resting for at least 5 min. Participants were advised to avoid smoking, caffeinated drinks, alcohol, and exercise for at least 30 min before measurement. Measurement was taken from the unclothed right arm of the person in a sitting position.

Systolic BP (SBP) and diastolic blood pressure (DBP) were measured using an Omron HEM-1300 electronic monitor. Height was measured without shoes using a standard right-angle device and a fixed measurement tape (to the nearest 0.1 cm). Body weight without heavy clothing was measured using a weight measurement device (V-body HBF-371.7, OMRON, Kyoto, Japan). Waistline circumference (WC) was measured in the midpoint between the lower rib and upper margin of the iliac crest, measured by a ruler tape with an insertion buckle at one end. WC was measured to the nearest 0.1 cm.

All study investigators and staff members including physicians, nurses, health care providers, and general practitioners were trained to be familiar with both the aims of the study and the specific tools and methods used.

### 2.3. Definitions

Hypertension (HT) was defined as having an SBP of ≥140 mm Hg and/or DBP of ≥90 mm Hg, or if the individual was on antihypertensive medication. Awareness was defined as whether they had a medical diagnosis of hypertension and treatment as whether they were receiving BP-lowering medications. Control was defined as an average SBP and DBP <140/90 mmHg.

Regarding education, participants were categorized into three levels: low education: primary and lower; middle: junior and senior high; and high: college and higher. Marital status was coded as single, married, or widow/divorced; occupational status as agriculture and stock-raising and nonagriculture and non-stock-raising; alcohol intake as current drinker (drinker was defined as consuming alcoholic beverage at least once per week in the past month) and nondrinker [10]; and cigarette consumption as current smoker (smokers were defined as participants who have smoked at least 20 packets of cigarettes in their lifetime and currently smoke cigarettes) and nonsmokers (participants who never smoked or smoked <20 packets of cigarettes in their entire lifetime) [10]. Body mass index (BMI) was calculated by dividing weight (in kilograms) by height (in meters) squared (kg/m^2^). To ensure comparability with other studies, our study incorporated the criteria recommended by the Working Group on Obesity in China (normal: BMI < 24.0 kg/m^2^; overweight: BMI 24.0–28.0 kg/m^2^; general obesity: BMI ≥ 28 kg/m^2^; and abdominal obesity: WC > 90 cm for men and >85 cm for women) and the WHO classifications for European (normal: BMI <25.0 kg/m^2^; overweight: BMI 25.0–30.0 kg/m^2^; general obesity: BMI >30 kg/m^2^; and abdominal obesity: WC > 102 cm for men and >88 cm for women). Urban and rural was divided according to the National Bureau of Statistics of the People's Republic of China published on the urban and rural regional code, the division of agricultural and stock-raising settings in accordance with the region's main source of economic income to distinguish [11].

### 2.4. Statistical Analysis

Data analysis was made for the overall study population by region and ethnicity. To enable comparisons between subgroups, results were adjusted by age and sex. Continuous variables were presented as mean (SD) deviations and were analyzed using ANOVA. Categorical variables were expressed as frequency (*n*) and proportion (%) and were analyzed using the Chi-square test. As for hypertension, multivariable logistic analysis was used to analyze the associated factors for prevalence, awareness, treatment, and control of hypertension; adjusted odds ratio (OR) with associated 95% confidence interval (95% CI) were calculated. For each dependent variable, the independent variables included in the multivariable logistic model were mainly the variables with statistical significance in the univariable analysis, which is set as *p* values ≤0.10. All statistical tests were two-tailed, and differences were considered statistically significant when the *p* value was <0.05. All statistical analyses were performed using SPSS 19.0 for Windows.

## 3. Results

### 3.1. Demographic Characteristics of Participants

**Figure 1:**
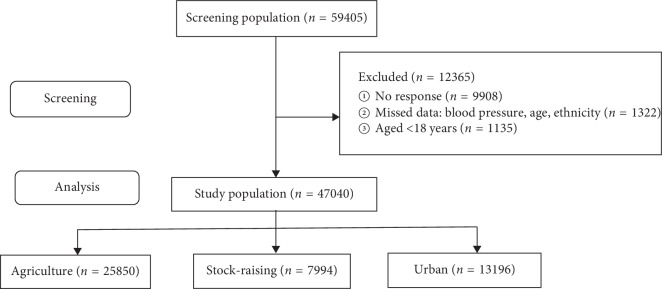
The process of recruiting the surveyed subjects including screening and analysis. 12,365 participants excluded due to missing data.

During door-to-door visit, 59405 subjects aged ≥15 years were invited for screening, of whom 49497 subjects (83.3% response rate) agreed, covering one third (34.5%, 49497/143289) of adult residents. 47040 subjects with complete data (2457 subjects were excluded; 1135 aged <18 years and 1322 with incomplete data) were analyzed with overall 48.5% women subjects (Figure 1). A total of 54.9% participants (*n* = 25850; aged 43.6 years) were enrolled from the agricultural setting, 17.0% (*n* = 7994; aged 42.0 years) from the stock-raising setting, and 28.1% (*n* = 13196; aged 45.9 years) were from the urban setting. Subjects from the urban setting were older than those from the stock-raising and agricultural settings. 77.4% subjects in the stock-raising setting were Kazakh ethnicity, and 53.4% in the urban setting were Han ethnicity; 43.3% in the agricultural setting were Han and 45.5% were Kazakh ethnicity. Subjects from the urban setting had significantly higher SBP than in those from the agricultural and stock-raising settings (Table 1).

### 3.2. Prevalence of Hypertension

Overall, 26.5% of participants aged ≥18 years (25.2% of men vs. 27.9% of women; *p* < 0.001) had hypertension. There were significant differences in age- and sex-standardized hypertension status for each subgroup (*p* < 0.001) (Table 2). The age- and sex-standardized prevalence of hypertension is 25.4% in the agricultural setting, 27.2% in the stock-raising setting, and 28.2% in the urban setting. The prevalence of hypertension increased with aging in both sex and in agricultural, stock-raising, and urban settings (*p* < 0.001). Kazakh subjects showed higher prevalence rate of hypertension than other ethnic groups (30.3% for Kazakh, 23.2% for Han, 28.8% for Uygur, and 27.8% for Mongolian).

### 3.3. Awareness, Treatment, and Control of Hypertension

Among those with hypertension, 64.6% (95% CI, 61.9%–67.1%) were aware of their condition and 44.5% (95% CI, 43.7%–45.2%) were taking medications to lower their BP, whereas only 15.3% (95% CI, 14.8%–15.9%) achieved BP control (Table 2).

The awareness, treatment, and control rates of hypertension were higher among women than men (67.1% vs. 61.9%, 47.8% vs. 41.2%, and 16.1% vs. 14.4%, respectively, *p* for all <0.001). The awareness and treatment rates were lower in patients from agricultural regions than in those from urban or stock-raising settings (awareness rate: 59.7% vs. 66.5% vs. 76.3%; treatment rate: 41.6% vs. 42.3% vs. 58.4%), whereas the control rate in patients from the stock-raising setting was lower than that of those from the urban and agricultural setting (13.8% vs. 15.2% vs. 15.8%, *p* < 0.05). The awareness, treatment, and control rates of hypertension were lower in Kazakh subjects than in other ethnic groups (*p* for all <0.001). The treatment was higher in participants with low education (*p*=0.022), whereas the control rate was lower (Table 2).

### 3.4. Multivariable Risk Assessment

After adjusting for age, gender, ethnicity, and regions, abdominal obesity (OR 1.30; 95% CI, 1.21–1.41), general obesity (OR 1.94; 95% CI, 1.75–2.15), cigarette consumption (OR 2.49; 95% CI, 2.20–2.81), and alcohol intake (OR 4.90; 95% CI, 4.33–5.53) were significantly associated with the presence of hypertension (*p* < 0.05) (Table 3, Supplementary [Supplementary-material supplementary-material-1]). Male gender and abdominal obesity were associated with lower awareness and treatment. Kazakh subjects, nonagricultural, and non-stock-raising occupation and lower education attainment were significantly associated with lower control of hypertension (*p* < 0.05) (Table 3, Supplementary Figures [Supplementary-material supplementary-material-1]–[Supplementary-material supplementary-material-1]).

## 4. Discussion

This study, to the best of our knowledge, was one of the largest hypertension screening project, seeking to explore the disparities in prevalence and management of hypertension in adult population from urban, agricultural, and stock-raising regions in Xinjiang, Northwest China. This study extended previous evidence to local agricultural and stock-raising settings that signify the current burden and status of hypertension in these specific settings. Main results encompassed that approximately 26.5% of residents aged ≥18 years in Xinjiang have hypertension, higher than the 23.1% of national average [10]. Hypertension treatment and control rates are approximate to the national average (treatment rate: 44.5% vs. 40.7%; control rate: 15.3% vs. 15.3%), whereas the gaps still exist between these rates and those of developed countries [12, 13].

Hypertension is the leading modifiable risk factor for CVD and stroke [1, 2]. However, no region and ethnicity in the current study has an overall control rate over 20%, which demonstrates that efforts to expand access to and improve the effectiveness of hypertension management are needed among multiethnic population in Northwest China. The strategies used, however, may vary with respect to the underlying regional and ethnic patterns revealed in this study. For example, our analysis showed the highest prevalence of hypertension in urban and stock-raising regions and in Kazakh and Uygur population and the lowest control rate in the stock-raising region and Kazakh. Thus, developing and practicing prioritized means of control for hypertension in these regions and ethnic groups are warranted.

Earlier epidemiological surveys reported large urban-rural gradients in Xinjiang, with lower prevalence of hypertension and lower rates of treatment and control in rural residents [14]. Currently, our study suggests that this gradient still exists locally. Nonetheless, this study further observes that the prevalence of hypertension is higher in stock-raising regions than agricultural regions, but with lower rates of control of hypertension in the stock-raising region compared with the agricultural setting. Background reasons may include the following: (1) Common antihypertensive agents are not readily available in many stock-raising regions. Furthermore, a large proportion of individuals could not afford, based on their household income, even if there are available agents in the stock-raising region [15, 16]. It also widely exists that hypertensive individuals often stop taking agents when BP control was achieved, which would also result in uncontrolled hypertension when checked later [17]. (2) Primary care physicians in the stock-raising region might have been less knowledgeable or experienced compared with tertiary hospital outpatients in urban settings. Furthermore, the village doctors region might have been entrenched in traditional prescription habits and lack knowledge or willingness to follow new guidelines due to obstacles in information exchange [17]. Therefore, current findings may highlight the need for developing a region-targeted hypertension education program to coordinate the efforts of detection, prevention, and treatment of hypertension in this region. In addition, considering the limited availability of antihypertensive agents and the limited affordability of locals, especially of nomads, exploring and promoting simplified antihypertensive algorithms might be the cost-effective and easy-to-master pathway for hypertension control.

Different prevalence and control of hypertension may also be attributable to different environmental exposures, ethnic-specific genetic susceptibility, and the interactions between gene and environment [18, 19]. Xinjiang is a multiethnicity-populated area. Kazakh and Uygur population have a mixture of 40% Asian ancestry and 60% European ancestry [20] and also have higher intake of salt and animal fat and proteins [21, 22], and stock-raising regions are characterized by few outdoor activities during long and cold winter, which may lead to a higher prevalence of hypertension than Han ethnicity. In addition, it is observed that Kazakh subjects, mostly leading nomadic or seminomadic life, have higher lifestyle-related risk factors for hypertension such as alcohol intake, cigarette consumption, and obesity and poor health awareness and poor health facility access due to higher illiteracy rates, language communication barriers, and mobile life [23], which may also contribute to their higher prevalence of hypertension and low control rates. In order to improve the prevention and control of hypertension in the stock-raising setting and Kazakhs, it is important to strengthen the promotion of health knowledge such as smoking cessation, alcohol restriction, and weight loss and to enhance primary care service. More importantly, Kazakh ethnicity is currently living in Kazakhstan, Uzbekistan, Russia, China, and Turkey, who still share lifestyle, dietary habits, and burden of hypertension [24]. Although a few studies reported higher prevalence of hypertension in this ethnic group [24], there are few epidemiological data on its associated factors. Therefore, results of the current study could extend to this specific population from other regions in terms of prevention of hypertension.

The present study highlights that alcohol intake, cigarette smoking, and obesity may increase the risk of hypertension in Xinjiang residents, consistent with previous literatures [25–28]. Most importantly, alcohol intake, compared to nondrinkers, was significantly related to increased prevalence of hypertension by 3.9 folds. Therefore, control of alcohol intake should be implemented as dominant (effective) measures or strategies to promote the prevention and management of hypertension in the setting.

Current analysis is strengthened by study subjects covering one third of locals and by their diversity in regions, age, sex, and ethnic groups, which makes them highly representative of locals [29]. However, this study contains some limitations. First, the population in this region prefer high salt intake, and we failed to collect salt intake of the population. Nonetheless, a survey is being conducted currently on salt intake in local population. Second, the cross-sectional nature of the study does not allow to get a cause-and-effect relationship between hypertension and related factors. However, it is also the common way of finding problems and providing clues for prevention. Third, this study did not investigate some information such as socioeconomic status and family income level, which may be related to the control of hypertension.

## 5. Conclusions

Existing disparities in hypertension and its management among regions and ethnic groups suggest its inadequate screening and treatment, especially in stock-raising regions and Kazakh populations. Control of alcohol intake, cigarette consumption, and weight and enhancement of primary care service should be at high priority of health promotion. Current results could also extend to Belt and Road countries such as Kazakhstan.

## Figures and Tables

**Table 1:** Baseline characteristics of the study population by region and sex.

Characteristic	Regions			Sex		Total
	Agriculture	Stock-raising	Urban	Men	Women	
Subjects, *n* (%)	25850 (55.0)	7994 (17)	13196 (28.1)	24229 (51.5)	22811 (48.5)	47040 (100)

Age (years)	43.6 (15.3)	42.0 (15.1)	45.9 (15.4)^b^	43.6 (15.3)	44.4 (15.4)	44.0 (15.3)

BMI, kg/m^2^, mean (SD)	24.6 (3.4)	24.3 (3.5)	24.7 (3.5)^b^	25.0 (3.3)	24.1 (3.6)	24.5 (3.4)

WC, cm, mean (SD)	90.2 (13.0)	89.6 (13.7)	90.5 (13.4)	92.0 (12.7)	88.2 (13.7)	90.2 (13.4)

Cigarette smoking, *n* (%)	4376 (16.9)	1430 (17.9)	2409 (18.3)	8063 (33.3)	152 (0.7)	8215 (17.5)

Alcohol drinking, *n* (%)	3827 (14.8)	1439 (18.0)	2216 (16.8)	7376 (30.4)	106 (0.5)	7482 (15.9)

Education status, *n* (%)						
Primary and lower	10631 (41.8)	2139 (26.9)	2080 (40.5)	7457 (36.9)	7393 (40.4)	14850 (38.6)
Elementary middle school	12127 (46.7)	5357 (67.3)	2428 (47.3)	10869 (53.8)	9043 (49.4)	19912 (51.7)
High school or above	2648 (10.4)	464 (5.8)	627 (12.2)	1866 (9.3)	1873 (10.2)	3739 (9.7)

Marital status, *n* (%)						
Single	3633 (14.1)	968 (12.1)	1487 (11.3)	3525 (14.5)	2563 (11.2)	6088 (12.9)
Married	21276 (82.3)	6927 (86.7)	11278 (85.5)	20197 (83.4)	19284 (84.5)	39481 (83.9)
Divorced	941 (3.6)	99 (1.2)	431 (3.3)^b^	507 (2.1)	964 (4.3)	1471 (3.2)

Occupation, *n* (%)						
Agricultural and husbandry	22466 (86.9)	6549 (91.9)	4317 (32.7)	17637 (72.8)	15695 (68.8)	33332 (70.9)
Nonagriculture or husbandry	3383 (13.1)	1445 (18.1)	8879 (67.3)	6591 (27.2)	7116 (31.2)	13707 (29.1)

Ethnicity, *n* (%)						
Han	11182 (43.3)	956 (12.0)	7042 (53.4)	9934 (41.0)	9246 (40.5)	19180 (40.8)
Kazakh	11767 (45.5)	6184 (77.4)	4160 (31.5)	11452 (47.3)	10659 (46.7)	22111 (47.0)
Uygur	628 (2.4)	112 (1.4)	1087 (8.2)	873 (3.6)	954 (4.2)	1827 (3.9)
Mongolian	1067 (4.1)	585 (7.3)	537 (4.1)	1049 (4.3)	1140 (5.1)	2189 (4.7)
Other	1206 (4.7)	157 (2.0)	370 (2.8)	921 (3.8)	812 (3.6)	1733 (3.7)

SBP, mmHg, mean (SD)	120.8 (16.0)	120.5 (17.3)	124.8 (17.6)^b^	122.9 (15.7)	120.8 (17.7)	121.9 (16.8)

DBP, mmHg, mean (SD)	76.4 (9.4)	76.4 (10.0)	76.5 (10.3)	77.3 (9.4)	75.5 (10.1)	76.5 (9.8)

BMI: body mass index; DBP, diastolic blood pressure; SBP, systolic blood pressure; SD, standard deviation; WC, waist circumference; *p* values for interaction comparing groups in regions:^a^<0.05; ^b^<0.0001. Regions: agriculture, stock-raising, and urban.

**Table 2:** Prevalence of hypertension and proportions who were aware, treated, and controlled in the study population.

		Prevalence (%) (95% CI)	Awareness (%) (95% CI)	Treatment (%) (95% CI)	Control (%) (95% CI)
Sex	Women	27.9 (24.5–30.3)	67.1 (64.3–69.8)	47.8 (44.3–49.5)	16.1 (14.3–18.7)
	Men	25.2 (23.3–28.1)	61.9 (58.2–64.2)	41.2 (38.7–43.7)	14.4 (12.2–16.5)

Age	18–24	1.7 (0.9–2.1)	6.3 (4.5–8.2)	—	—
	25–34	3.5 (1.2–8.0)	21.6 (19.7–24.2)	6.9 (4.2–9.7)	2.3 (0.8–4.6)
	35–44	16.6 (12.4–19.8)	31.9 (29.3–34.4)	16.3 (13.4–19.8)	5.3 (3.1–7.6)
	45–54	33.9 (29.1–37.4)	58.1 (54.6–62.1)	38.0 (35.2–40.6)	12.7 (9.2–15.1)
	55–64	53.2 (49.1–58.2)	74.2 (70.4–77.9)	52.2 (49.6–55.9)	18.1 (15.6–21.4)
	≥65	66.3 (62.3–70.1)	84.8 (80.9–87.4)	63.6 (60.1–66.9)	22.2 (18.8–25.9)

Regions	Agriculture	25.4 (23.6–27.1)	59.7 (57.1–61.6)	41.6 (39.9–43.2)	15.8 (14.1–17.4)
	Stock-raising	27.2 (25.3–29.0)	76.3 (74.5–78.1)	58.4 (56.8–60.1)	13.9 (12.2–15.7)
	Urban	28.2 (26.4–30.2)	66.5 (64.8–68.2)	42.3 (40.5–44.0)	15.4 (13.8–17.2)

Ethnicity	Han	23.2 (21.8–24.6)	66.5 (65.1–67.9)	47.0 (45.5–48.4)	17.5 (16.1–19.0)
	Kazakh	30.3 (28.9–32.7)	62.3 (60.9–64.8)	41.5 (38.9–44.1)	12.6 (10.2–15.1)
	Uygur	28.8 (26.4–31.2)	70.4 (67.0–73.9)	44.8 (42.3–47.2)	19.9 (17.5–22.3)
	Mongolian	27.8 (25.4–30.2)	65.3 (63.0–67.8)	45.1 (42.7–47.5)	14.8 (12.3–17.2)
	Others	23.0 (21.6–24.4)	59.8 (57.4–62.2)	45.4 (43.9–47.8)	22.8 (20.2–25.3)

Education status	Primary and lower	29.0 (26.6–32.5)	61.5 (58.7–64.3)	43.7 (40.9–45.3)	13.6 (11.2–15.9)
	Elementary middle school	20.5 (18.1–22.9)	66.3 (64.6–69.1)	46.2 (43.8–49.1)	17.2 (15.7–19.5)
	High school or above	12.6 (10.3–14.9)	62.5 (59.9–65.3)	41.4 (38.7–44.2)	24.5 (22.1–27.0)

All		26.5 (23.7–28.1)	64.6 (61.9–67.1)	44.5 (41.7–47.2)	15.3 (12.8–16.9)

Adjusted age and sex; control is defined as BP < 140/90 mmHg.

**Table 3:** Factors associated with hypertension from multiple logistic regression.

Variables	Stratification	Prevalence OR (95% CI)	Awareness OR (95% CI)	Treatment OR (95% CI)	Control OR (95% CI)
Gender	Women	1 (reference)	1 (reference)	1 (reference)	1 (reference)
	Men	0.88 (0.82–0.94)	0.77 (0.67–0.87)	0.80 (0.72–0.88)	0.94 (0.83–1.06)

Age	18–44	1 (reference)	1 (reference)	1 (reference)	1 (reference)
	45–60	4.95 (4.61–5.31)	4.71 (4.10–5.43)	4.15 (3.57–4.82)	3.08 (2.44–3.90)
	60+	12.29 (11.28–13.38)	11.55 (9.82–13.57)	8.26 (7.05–9.68)	3.90 (3.07–4.94)

Regions	Agriculture	1 (reference)	1 (reference)	1 (reference)	1 (reference)
	Stock-raising	0.96 (0.79–1.23)	2.38 (2.04–2.78)	1.98 (1.74–2.25)	0.98 (0.83–1.15)
	Urban	1.63 (1.47–1.79)	1.03 (0.86–1.23)	1.05 (0.89–1.25)	0.82 (0.66–1.03)

Ethnicity	Han	1 (reference)	1 (reference)	1 (reference)	1 (reference)
	Kazakh	1.68 (1.57–1.80)	0.90 (0.88–1.12)	1.10 (0.99–1.23)	0.58 (0.51–0.67)
	Uighur	0.94 (0.76–1.16)	1.17 (0.77–1.79)	1.16 (0.81–1.65)	1.23 (0.84–1.80)
	Mongolian	1.42 (1.23–1.64)	1.01 (0.77–1.32)	1.08 (0.86–1.36)	0.78 (0.59–1.03)
	Other	0.78 (0.66–0.93)	0.81 (0.59–1.13)	1.12 (0.83–1.50)	1.03 (0.74–1.44)

Education status	Primary and lower	1 (reference)	1 (reference)	1 (reference)	1 (reference)
	Elementary middle school	0.62 (0.58–0.66)	1.20 (1.07–1.36)	0.98 (0.89–1.10)	1.29 (1.13–1.47)
	High school or above	0.33 (0.29–0.39)	0.87 (0.63–1.20)	0.74 (0.54–1.02)	1.38 (0.96–2.00)

Occupation	Agriculture or husbandry	1 (reference)	1 (reference)	1 (reference)	1 (reference)
	Nonagriculture or nonhusbandry	2.24 (2.07–2.43)	1.08 (0.94–1.23)	1.01 (0.89–1.13)	0.64 (0.54–0.75)

Marital status	Single	1 (reference)	1 (reference)	1 (reference)	1 (reference)
	Married	2.55 (2.14–3.03)	1.99 (1.30–3.07)	2.76 (1.57–4.85)	1.05 (0.55–1.99)
	Divorced	3.55 (2.84–4.44)	2.82 (1.74–4.59)	3.14 (1.74–5.67)	1.65 (0.85–3.23)

BMI (WGOC)	Normal	1 (reference)	1 (reference)	1 (reference)	1 (reference)
	Overweight	1.30 (1.20–1.41)	1.43 (1.23–1.65)	1.39 (1.22–1.59)	1.23 (1.04–1.46)
	Obesity	1.94 (1.75–2.15)	2.73 (2.27–3.27)	1.20 (1.70–2.34)	1.44 (1.17–1.77)

BMI (WHO)	Normal	1 (reference)	1 (reference)	1 (reference)	1 (reference)
	Overweight	1.36 (1.27–1.46)	1.38 (1.22–1.57)	1.20 (1.07–1.34)	1.15 (0.99–1.33)
	Obesity	2.11 (1.87–2.37)	3.17 (2.57–3.92)	1.93 (1.63–2.29)	1.45 (1.18–1.79)

WC (WGOC)	Normal	1 (reference)	1 (reference)	1 (reference)	1 (reference)
	Abdominal obesity	1.30 (1.21–1.41)	0.80 (0.70–0.93)	0.85 (0.75–0.97)	0.89 (0.75–1.05)

WC (WHO)	Normal	1 (reference)	1 (reference)	1 (reference)	1 (reference)
	Abdominal obesity	1.32 (1.22–1.41)	0.996 (0.87–1.14)	1.03 (0.92–1.16)	0.96 (0.83–1.12)

Cigarette smoking	No	1 (reference)	1 (reference)	1 (reference)	1 (reference)
	Yes	2.49 (2.20–2.81)	4.88 (3.66–6.50)	2.64 (1.94–3.58)	1.79 (1.23–2.60)

Alcohol drinking	No	1 (reference)	1 (reference)	1 (reference)	1 (reference)
	Yes	4.90 (4.33–5.53)	11.37 (8.58–15.07)	8.04 (5.84–11.05)	2.89 (1.98–4.23)

BMI: body mass index; DBP, diastolic blood pressure; SBP, systolic blood pressure; SD, standard deviation; WC, waist circumference; WGOC: Working Group on Obesity in China.; OR: odd ratio; CI: confidence interval.

## Data Availability

Materials included in the manuscript, including all relevant raw data, will be made freely available to any researchers who wish to use them for noncommercial purposes, while preserving any necessary confidentiality and anonymity.

## Supplementary Materials

Supplementary Materials Factors associated with prevalence, awareness, treatment, and control of hypertension from multiple logistic regressions: Supplementary Figure 2: hypertension, Supplementary Figure 3: awareness, Supplementary Figure 4: treatment, Supplementary Figure 5: control.
