# Supplementary material for: Prevalence, Awareness, Treatment, and Control and Related Factors of Hypertension in Multiethnic Agriculture, Stock-Raising, and Urban Xinjiang, Northwest China: A Cross-Sectional Screening for 47000 Adults
Source: Int J Hypertens. 2019 Nov 3;2019:3576853. doi: 10.1155/2019/3576853 (PMC6875381; doi:10.1155/2019/3576853)
Supplement: Supplementary Materials — Factors associated with prevalence, awareness, treatment, and control of hypertension from multiple logistic regressions: Supplementary Figure 2: hypertension, Supplementary Figure 3: awareness, Supplementary Figure 4: treatment, Supplementary Figure 5: control. [file 3576853.f1.pdf]

Factors associated with prevalence, awareness, treatment and control of hypertension from multiple logistic regressions: Supplementary-Fig 2: Hypertension, Supplementary-Fig 3: Awareness, Supplementary-Fig 4: Treatment, Supplementary-Fig 5: Control.

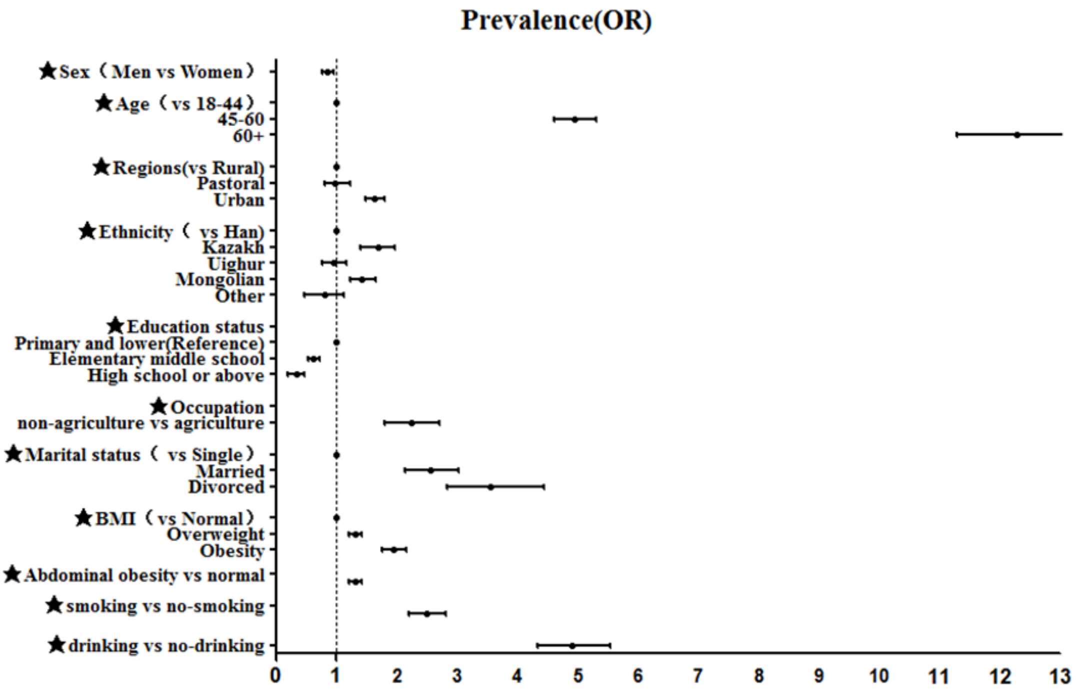

Fig 2. Factors associated with HT From multiple logistic regression Emin adults

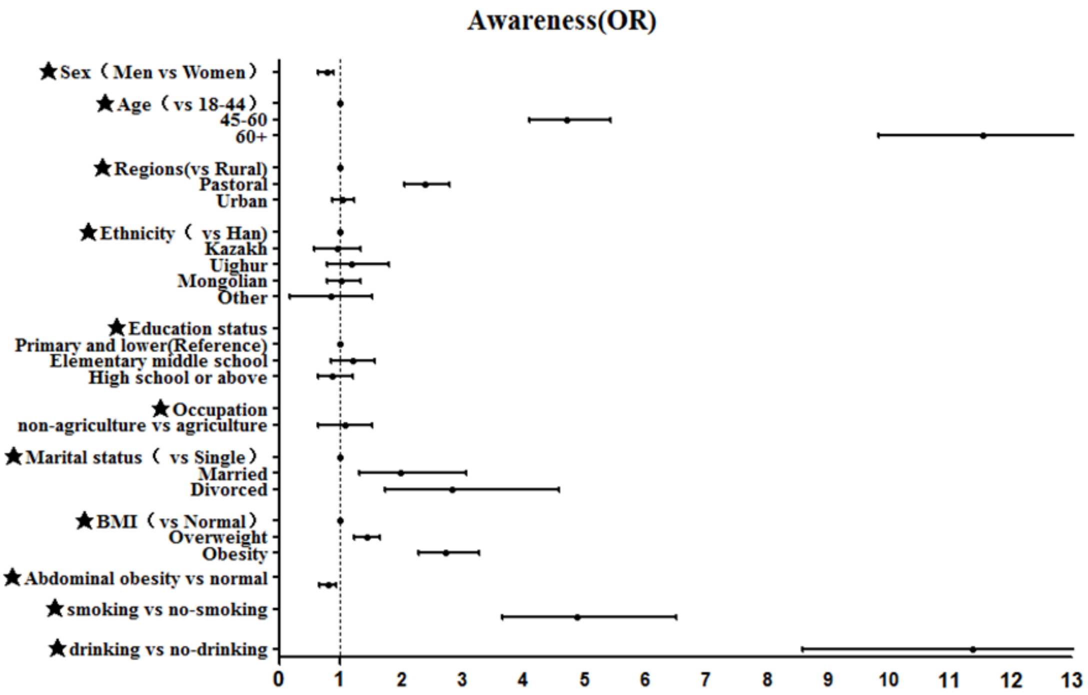

Fig 3. Factors associated with awareness of HT From multiple logistic regression Emin adults

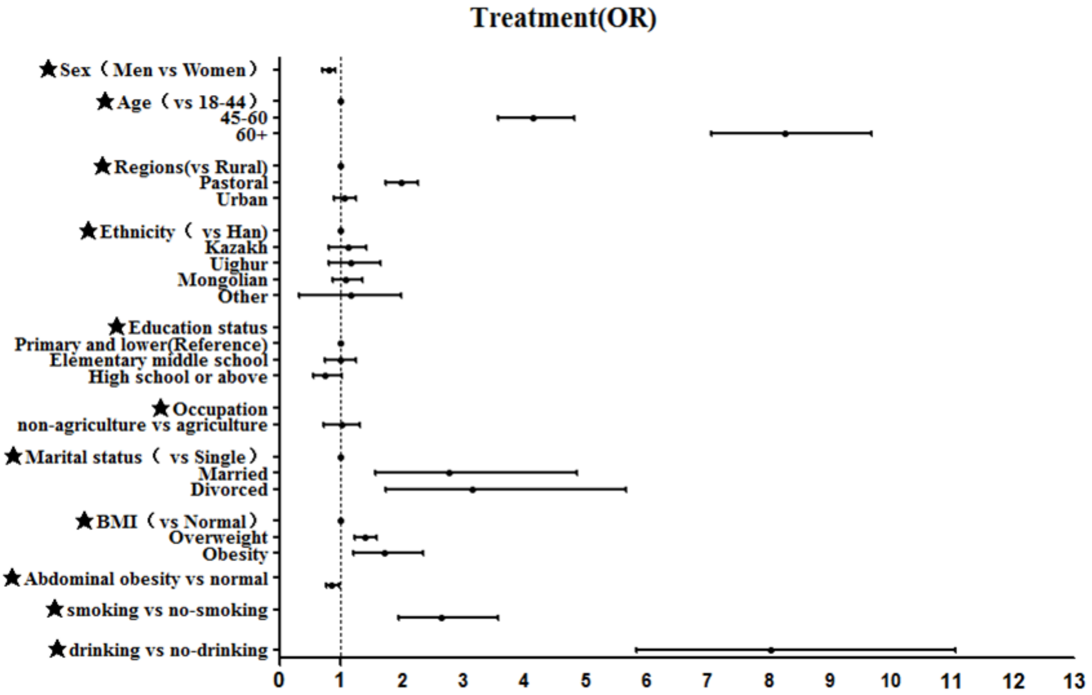

Fig 4. Factors associated with treatment of HT From multiple logistic regression Emin adults

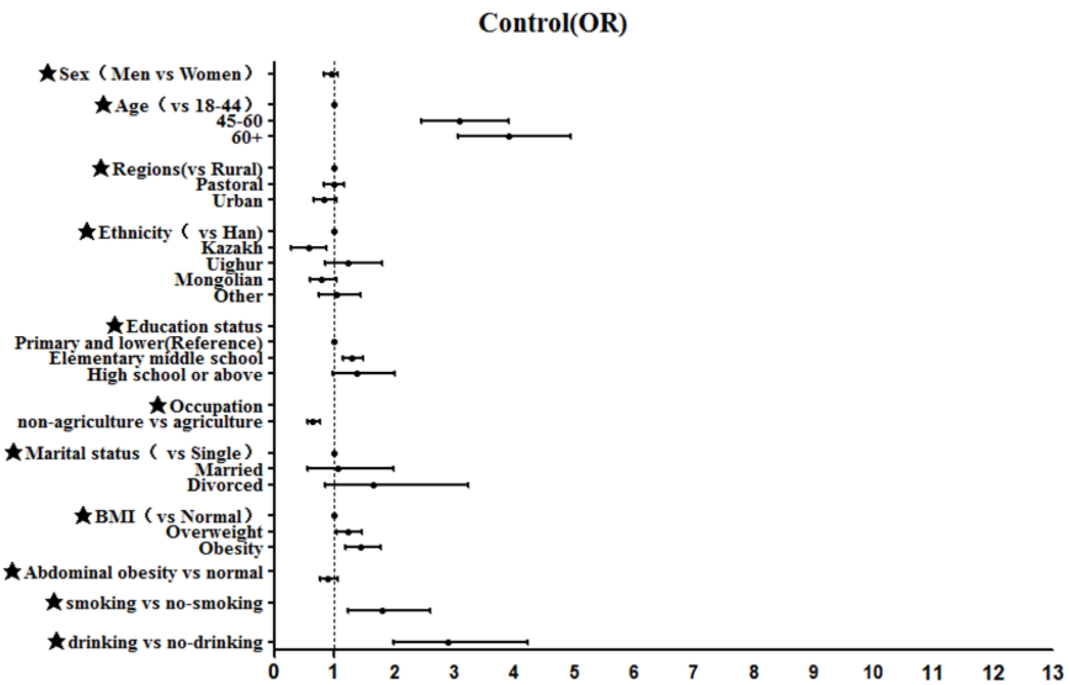

Fig 5. Factors associated with control of HT From multiple logistic regression Emin adults
